# Using bioinformatics technology to mine the expression of serum exosomal miRNA in patients with traumatic brain injury

**DOI:** 10.3389/fnins.2023.1145307

**Published:** 2023-04-18

**Authors:** Xintao Huang, Xinjuan Xu, Ce Wang, Yi Wang, Yajun Yang, Tianle Yao, Rui Bai, Xile Pei, Feirong Bai, Panpan Li

**Affiliations:** ^1^Department of Neurosurgery, First Hospital of Shanxi Medical University, Taiyuan, Shanxi, China; ^2^Department of Neurosurgery, Shanxi Cardiovascular Hospital, Taiyuan, Shanxi, China; ^3^The First School of Clinical Medicine, Shanxi Medical University, Taiyuan, Shanxi, China

**Keywords:** traumatic brain injury, exosome, microRNA (miR), next-generation sequencing (NGS), bioinformatics analysis

## Abstract

**Introduction:**

Traumatic brain injury (TBI) is considered the most common traumatic neurological disease, is associated with high mortality and long-term complications, and is a global public health issue. However, there has been little progress in serum markers for TBI research. Therefore, there is an urgent need for biomarkers that can sufficiently function in TBI diagnosis and evaluation.

**Methods:**

Exosomal microRNA (ExomiR), a stable circulating marker in the serum, has aroused widespread interest among researchers. To explore the level of serum ExomiR after TBI, we quantified ExomiR expression levels in serum exosomes extracted from patients with TBI using next-generation sequencing (NGS) and explored potential biomarkers using bioinformatics screening.

**Results:**

Compared with the control group, there were 245 ExomiR (136 up-regulated and 109 down-regulated) in the serum of the TBI group that changed significantly. We observed serum ExomiRs expression profiles associated with neurovascular remodeling, the integrity of the blood-brain barrier, neuroinflammation, and a cascade of secondary injury, including eight up-regulated ExomiRs (ExomiR-124-3p, ExomiR-137-3p, ExomiR-9-3p, ExomiR-133a-5p, ExomiR-204-3p, ExomiR-519a-5p, ExomiR-4732-5p, and ExomiR-206) and 2 down-regulated ExomiR (ExomiR-21-3p and ExomiR-199a-5).

**Discussion:**

The results revealed that serum ExomiRs might become a new research direction and breakthrough for the diagnosis and pathophysiological treatment of patients with TBI.

## 1. Introduction

Traumatic brain injury (TBI) is a disease of the most complex organ. The fatality rate associated with TBI has been increasing annually and has become a critical public health issue worldwide, with nearly 90% of post-injury deaths occurring in low- and middle-income countries based on a survey by the World Health Organization (WHO) (Rubiano et al., 2015; Kamal et al., 2016; Dewan et al., 2019). Epidemiologically, the mortality rate of TBI is approximately 13 cases per 100,000 people annually in China (Jiang et al., 2019). From 2001 to 2016, approximately 120,000 patients suffered from acute TBI, according to 18 retrospective clinical studies in China (Jiang et al., 2007; Xu et al., 2016; Cheng et al., 2017; Li et al., 2017). Research has greatly clarified TBI’s mechanisms, involving a primary insult caused by direct biomechanical forces and a secondary insult, resulting in brain damage and even death (Werner and Engelhard, 2007). Following a primary mechanical injury in TBI, the literature suggests the presence of a delayed secondary injury involving various neuroinflammatory changes. Several signaling molecules and metabolic derangements disrupt the blood-brain barrier (BBB) from hours to days following a TBI, leading to the extravasation of immune cells and cerebral edema. Secondary brain injury, which is the focus of current research and clinical treatment, involves many molecular mechanisms, including the neuroinflammatory response, apoptosis, oxidative damage, BBB damage, and regulation of gene expression (Cornelius et al., 2013; Crupi et al., 2020).

Exosomes are disc-shaped vesicles of 30–150 nm diameter secreted by various cells and are widely distributed in the blood, urine, saliva, cerebrospinal fluid, and other extracellular fluids. Intracellular vesicles are formed by endocytosis, which fuses with the cell membrane and is secreted to form extracellular vesicles. They mediate cellular communication by transporting proteins, lipids, miRs, messenger RNA (mRNA), and DNA from the parent cell to the recipient cell (Zhang et al., 2015). Therefore, they may serve as diagnostic and prognostic serum biomarkers. After trauma, brain-cell-derived exosomes containing miR, mRNA, or DNA can cross the BBB to the peripheral blood. MiRs are endogenous non-coding RNAs with a length of about 22 nucleotides that regulate gene expression by mediating translation inhibition or mRNA degradation. Exosomal miRs are involved in the occurrence and development of a variety of central nervous system diseases, such as TBI (Ghai et al., 2020), spinal cord injury (Bao et al., 2018), stroke (Zhou et al., 2018), and neurodegenerative diseases (Nie et al., 2020). Therefore, exosomal miRs might be necessary to transfer information, regulate signal pathways, and provide a new diagnosis and treatment strategy for central nervous system (CNS) diseases.

In this study, exosomes and exosomal miRs in the peripheral serum of patients with TBI were isolated, and high-throughput sequencing was used to study the exosomal miRNA expression changes in patients with TBI with different degrees of injury. This study used bioinformatics analysis techniques to predict the targets of differential genes, revealing potential miRs in peripheral blood exosomes after TBI. Related research deepened people’s understanding of the molecular mechanism after TBI and provided potential intervention sites after pathological changes. The expression of exosomal miR in patients with TBI provides many TBI-related biomarkers for clinical research and improves the accuracy of the condition judgment and prognostic evaluation of patients with TBI.

## 2. Materials and methods

### 2.1. Patient recruitment and serum sample collection

The inclusion criteria for the patients were as follows: (1) the patient was between the ages of 18–80 years, (2) the patient got admitted within 24 h of the TBI event, and (3) patient with no surgical treatment. In addition, the following patients were excluded: (1) Complications with other system injuries (including the heart, liver, and lungs, etc.) and long bone fractures (upper limbs or femurs); and (2) Central nervous system disease, cardiovascular and cerebrovascular disease, and blood system disease have been diagnosed or documented with other medical histories.

A coagulation tube collected approximately 8–10 mL of upper arm venous blood from patients at 24 hospitals before surgery, mixed by inverting, allowed to stand for 2 h, and centrifuged at 1000 *g* at 4°C for 15 min. The supernatant was extracted, aliquoted into RNase-free 1 mL EP tubes, and stored at −80°C.

### 2.2. Isolation of exosomes derived from patients with TBI

Frozen exosome serum samples extracted by the high-speed centrifugal method were thawed, melted in a 25°C water bath, and transferred to a centrifuge tube. Impurities were removed from the samples by centrifugation at 4°C, 3000 × g for 10 min and then again for 20 min at 10,000 × g. The impurities and cell debris were transferred to a new centrifuge tube; 4 mL phosphate buffer saline (PBS) and 1 mL Exo extraction purification reagent (Blood Pure Exo Solution, BPS) were added and mixed through a vortex oscillator for 1 min, at 2–8°C for 2 h; Centrifuge tube with the mixture was centrifuged at 10,000*g* for 60 min, the supernatant was discarded, and the precipitate was rich with exosome particles (note: as clean as possible); Then, 0.2 mL 1 × PBS was added to the sediment evenly after uniform suspension (recommended PBS liquid added volume: initial sample volume = 1:5); this was transferred to a new 1.5 mL centrifuge tube and centrifuged for 2 min at 4°C 12000 × g. The supernatant was moved to the EPF (Exosome Purification Filter) column upper chamber after centrifugation at 4°C, 3000 × g for 10 min and then purified using Exo particles. Purified Exo was stored in 0.05–0.1 mL portions in an −80 °C refrigerator for future experiments.

### 2.3. Identification of exosomes derived from patients with TBI

The morphological characteristics of exosomes were visualized using transmission electron microscopy (TEM; JEOL-1230; Tokyo, Japan). Briefly, 30 μL of exosome samples were placed on a sheet of parafilm, and a 100-mesh copper grid was transferred to drops of exosomes with forceps for 10 min. Phosphotungstic acid was then used to stain the grid for 15 s and dried at 23°C and 30°C. The size of exosomes was detected by nanoparticle tracking analysis (NTA) using ZetaView PMX110 (Particle Metrix, Meerbusch, Germany) and its accompanying software. Isolated exosome samples were appropriately diluted using 1X PBS buffer (Biological Industries, Israel) to measure particle size and concentration. The levels of multiple exosome markers (CD63 and TSG101) were measured by western blotting.

### 2.4. miR expression in exosomes of patients with TBI

Total RNA was extracted from serum exosomes with TRIzol (Invitrogen, Carlsbad, CA, USA) reagent and was further purified.

### 2.5. Bioinformatic analyses

#### 2.5.1. Exosomal miR library construction and sequencing

The TruSeq Small RNA Prep Kit (RS-122-2002, Illumina, USA) was used to construct libraries according to the manufacturer’s protocol. The optimal loading amount was selected for sequencing on the Illumina platform.

#### 2.5.2. Filtering and miR mapping

After sequencing, clean data were filtered from raw reads using the following criteria: (a) 30% base quality < 20; (b) read length < 18 nucleotides (nt), >36 nt; (c) adaptor sequence; (d) align to the reference genome, remove unmatched sequences; (e) align to ncRNA (non-coding RNA) in Rfam, remove snRNA (small nuclearRNA), snoRNA (small nucleolar RNA), tRNA (Transfer RNA), etc.; and (f) remove repetitive sequences. The Rfam (Kalvari et al., 2018) genome sequence was compared with clean reads after de-redundancy to find known miRs and new miRs, siRNA, piRNA, and other sRNA molecules, and obtain their respective expression levels. Differential miRNA expression analysis was performed for each sample.

#### 2.5.3. Differential expression analysis of exosomal miR

We performed statistical analysis on the expression level of exosome miR in each sample. We used | log2FC| (| log2Fold Change|) > 1.0 and *P* < 0.05 as the screening criteria and DESeq software to screen for differential expression. Based on the analysis results, the common and unique differences in genes between the TBI and control groups were screened.

#### 2.5.4. Predict the target genes of miRs with significant differential expression

Targets can include Mir Walk (Sticht et al., 2018), Mirta Base, and Miranda to predict the target genes of miRs with significant differential expression.

#### 2.5.5. GO function annotation and KEGG pathway enrichment analysis

For target genes with differentially expressed miRNAs, we used the DAVID (Huang et al., 2009) website to perform GO (Gene ontology) function annotation analysis and KEGG (Kyoto Encyclopedia of Genes and Genomes) pathway enrichment analysis to screen enrichment analysis items and pathway information. Statistical significance was set at *P* < 0.05.

#### 2.5.6. Construct a protein-protein interaction (PPI) network map

A PPI diagram of differentially expressed miR target genes was constructed using String (version 11.5) (Szklarczyk et al., 2021). A cystoscope was used to visualize the results.

### 2.6. Statistical analysis

The serum expression levels of candidate exosomal miRs in patients and control subjects were analyzed using the non-parametric Mann–Whitney test. All statistical analyses were performed using SPSS 22.5 software. *P* < 0.05 was considered a statistically significant difference. The study process is shown in Supplementary Table 1.

## 3. Results

### 3.1. Basic information and general characteristics of the patient

A total of 14 patients (male 12, female 2, mean age 58.4 ± 14.9) with TBI were admitted to our department from November 1, 2019, to January 31, 2020, age ranging from 30 to 80 years, and 6 matched healthy controls (1 female, 5 males, mean age 58.5 ± 16.4) were used for our study.

The patients with TBI were divided into two groups according to the Glasgow Coma Scale (GCS) on admission: mild TBI and moderate TBI (mTBI) (GCS = 9–15): 7 cases (35%); severe TBI (sTBI) group (GCS ≤ 8): 7 cases (35%); and control group, 6 cases (30%) (Table 1).

**TABLE 1:** Basic information and general characteristics of the patients.

Variable	Group A = sTBI (*n* = 7)	Group B = mTBI (*n* = 7)	Control (*n* = 7)
Age (years)	64.14 (39–81)	52.71 (38–81)	58.5 (30–80)
Male sex (M/F ratio)	6:1	5:2	4:2
Mechanism of injury			
Falls	2 (28.57%)	3 (43%)	
Exposure to mechanical forces	2 (28.57%)	1 (14.25%)	
Transport accidents	3 (42.86%)	2 (28.5%)	
Assaults	0	1 (14.25%)	
Initial vital signs			
GCS	5.71 ± 2.36	12 ± 2.38	
SBP	132.71 ± 14.56	117.56 ± 16.47	
HR	80.43 ± 9.91	84 ± 13.34	
Hemoglobin	49.2 ± 35.54	33.14 ± 21.08	
Initial pathology results			
HB	127.43 ± 12.45	134.71 ± 15.85	
PLT	151 ± 48.17	172.29 ± 39.27	
WBC	15.69 ± 2.75	13.44 ± 2.09	
Injury type			
SDH	2 (28.5%)	2 (28.5%)	
EDH	1 (14.25%)	0	
SAH	4 (57.14%)	4 (57.14%)	
Intracerebral hematoma	3 (42.86%)	4 (57.14%)	
Fracture of skull	4 (57.14%)	5 (71.43%)	
Cerebral contusion and laceration	6 (85.71%)	6 (85.71%)	
Marshall CT scan score (% of pts)			
II	13	43	
III	0	14	
IV	29	0	
V	29	29	
VI	29	14	

SBP, systolic blood pressure; HR, heart rate; PLT, platelet count; HB, hemoglobin; WBC, white blood cell; SDH, subdural hematoma; EDH, epidural hematoma; SAH, subarachnoid hemorrhage.

### 3.2. Identification of exosomes

**FIGURE 1:**
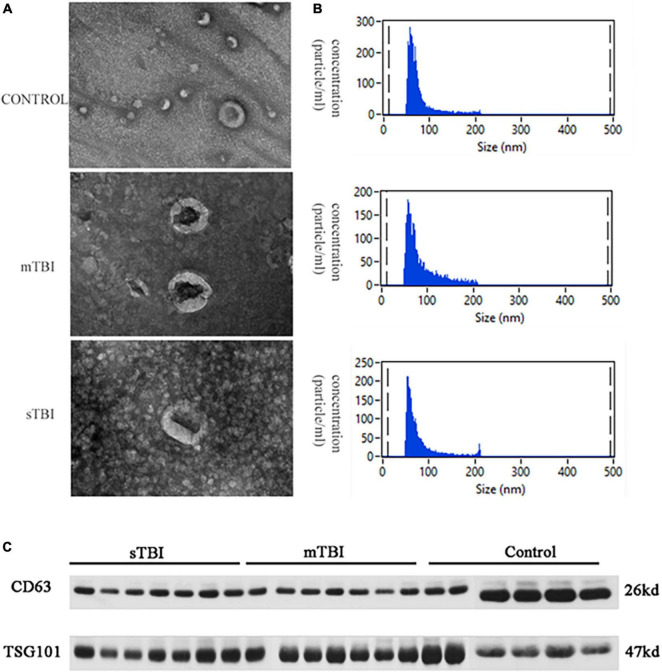
The characterization of the isolation exosome. **(A)** The TEM image shows the morphology of the exosomes. **(B)** NTA measured the size distribution of exosomes. **(C)** Western blotting of the exosomal membrane markers CD63 and TSG101.

Serum was extracted from the peripheral blood of patients with TBI and the control group. Exosomes in serum were separated and extracted by high-speed centrifugation. After quantification using the BCA (bicinchoninic acid) protein analysis kit, exosome morphology was observed by TEM. As shown in Figure 1A, the particles isolated from the control group, sTBI, and mTBI microglia had a typical sphericity-shaped morphology and 50–200 nm diameters. The concentration and size distribution of the isolated particles were analyzed using NTA (Figure 1B). Furthermore, western blot analysis showed that characteristic exosome biomarkers, including CD63 and TSG101, were expressed in particles isolated from the TBI and control groups (Figure 1C). The above results were confirmed from the serum as an external body by the size of the extracellular vesicle, expression of the morphology, and protein markers.

### 3.3. Differential gene expression in patients with TBI

#### 3.3.1. Identification of differentially expressed genes from exosomal miRs in patients with TBI

**FIGURE 2:**
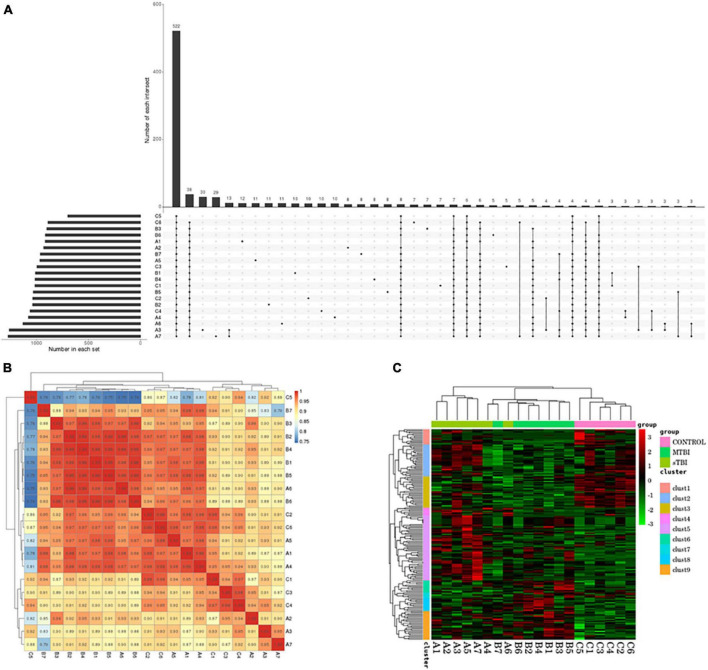
**(A)** UpSet plots obtained from each sample identification: the number in each set represents the number of all miRs identified by each sample; the number of each intersection represents the number of common miRs identified by multiple samples; and the abscissa one point represents the number of unique miRs identified by that sample item; multiple abscissa point connections represent the number of common miRs identified by multiple samples of the connection. **(B)** The correlation of commonly expressed genes between TBI and the control group, analyzed using Pearson’s correlation analysis: the sample is clustered on the left and upper sides, the sample names and colored squares represent the correlation between the two samples. **(C)** Differentially expressed miR clustering: Transsternal indicates miR; each column is one sample; red indicates high miR; green indicates low miR.

Statistical and differential expression analyses of miRs identified in each sample were performed according to the expression data of each sample. To understand the changes in serum exosomal miRs in patients with TBI with different degrees of injury at the genetic level. Figure 2A shows that in all samples in the TBI and control groups, 522 miRs were identified in total.

Pearson’s correlation coefficient analysis was used to analyze the correlation of miR expression levels between samples to test the experiment’s reliability and whether the sample selection was reasonable (Figure 2B).

The Euclidean method was used to calculate the distance to assess the correlation of differentially expressed miRs between samples. The hierarchical clustering-longest distance method (complete linkage) was used for clustering. Clustering was performed based on the expression level of the same miR in different samples and the expression pattern of different miRs in the same sample (Figure 2C).

#### 3.3.2. Exosomal miR differentially expressed analysis

**FIGURE 3:**
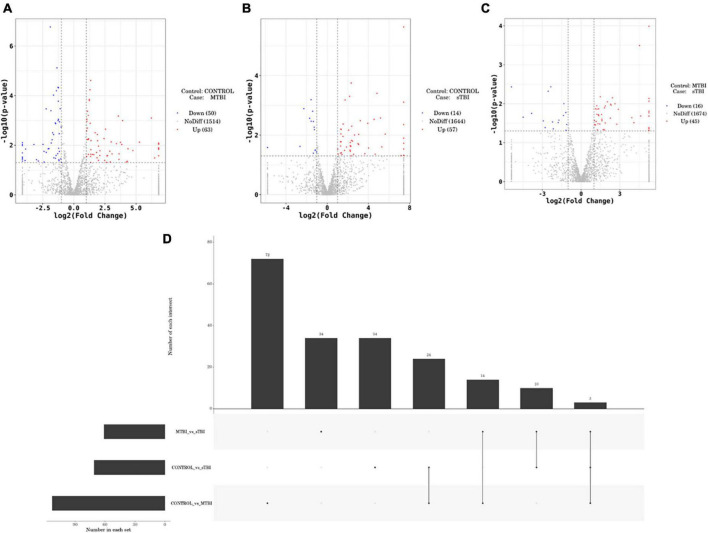
Volcano plots of differentially expressed genes comparing mTBI and control, sTBI and control, and sTBI and mTBI. **(A)** Compared to the control group, the differentially expressed miRs of mTBI. **(B)** Compared with the control group, the differentially expressed miRs of sTBI. **(C)** Compared with the mTBI group, the differential expression of miR in the sTBI group. The abscissa is |log2FoldChange|, and the ordinate is −log10 (*p*-value). The two vertical dashed lines in the figure are the 2-fold expression difference threshold, and the horizontal dashed line is the *P* = 0.05 threshold. Red dots indicate up-regulated genes in this group; blue dots indicate down-regulated genes in this group; and grey dots indicate non-significantly differentially expressed genes. **(D)** Different analyses of the groups revealed a common unique differential miR.

DESeq software (version 1.18.0, Anders and Huber, 2010) was used to screen differentially expressed miRs and perform correlation analysis. In total, 191 differentially expressed miRs were screened. Compared with the control group, 113 miRs were differentially expressed in the mTBI group, of which 50 were down-regulated, and 63 were up-regulated (Figure 3A and Table 2). In contrast, 71 differentially expressed miRs were observed in the sTBI group, including 14 miRNAs that were down-regulated and 57 that were up-regulated (Figure 3B and Table 3). Compared with the mTBI group, 61 miRs were differentially expressed in the sTBI group, 16 miRs were down-regulated, and 45 miRs were up-regulated (Figure 3C and Table 4). Compared to the control group, the TBI group had 27 differentially expressed miRs (Figure 3D and Table 5). Three differentially expressed miRs (ExomiR-206, ExomiR-133a-3p, and ExomiR-549a-3p) co-occurred after pairwise control among the three groups (Figure 3D and Table 6).

**TABLE 2:** Control-mTBI.

Down-regulated miRs	log2FoldChange	*P*-value
ExomiR-1185-1-3p	−1.29450722	0.021859964
ExomiR-199a-5p	−1.245428592	0.000167891
ExomiR-21-3p	−1.231346788	4.82376E-05
ExomiR-301a-5p	−1.158499054	0.017849977
ExomiR-33b-5p	−2.396267017	0.017751943
ExomiR-374b-3p	−1.486177978	0.001249994
ExomiR-4433b-5p	−1.039552586	0.035479216
ExomiR-6852-5p	−1.632485465	9.5383E-05
**Up-regulated miRs**	**log2FoldChange**	***P*-value**
ExomiR-6875-5p	Inf	0.00902925
ExomiR-124-3p	6.495166389	0.032432744
ExomiR-4745-5p	6.23036245	0.0007964
ExomiR-137-3p	5.269872718	0.007493811
ExomiR-9-3p	4.355155062	0.013841228
ExomiR-133a-5p	3.848727246	0.009333523
ExomiR-3154	3.574760228	0.000667226
ExomiR-708-5p	2.997830054	0.007325137
ExomiR-208b-3p	2.63431488	0.049079872
ExomiR-499a-5p	2.48243991	0.040721098
ExomiR-519a-5p	2.087808973	0.025738171
ExomiR-4683	1.865183316	0.031947772
ExomiR-6780a-5p	1.826426376	0.019512214
ExomiR-204-3p	1.355669	0.022924106
ExomiR-1304-5p	1.348109171	0.049076138
ExomiR-4732-5p	1.29673322	5.78117E-05

**TABLE 3:** Control-sTBI.

Down-regulated miRs	log2FoldChange	*P*-value
ExomiR-374a-3p	−1.565782504	0.000644216
ExomiR-6852-5p	−1.554712377	0.003402456
ExomiR-4433b-5p	−1.341900632	0.003480341
ExomiR-21-3p	−1.242727793	0.006524039
ExomiR-33b-5p	−2.638578801	0.023814363
ExomiR-1185-1-3p	−1.170118592	0.032553239
ExomiR-199a-5p	−1.03407065	0.03675369
ExomiR-301a-5p	−1.344373369	0.040374543
**Up-regulated miRs**	**log2FoldChange**	***P*-value**
ExomiR-3154	4.82824325	0.000393672
ExomiR-204-3p	1.738337751	0.000656784
ExomiR-4683	2.388800701	0.004983982
ExomiR-499a-5p	1.323962723	0.006285746
ExomiR-4745-5p	5.627512215	0.009240171
ExomiR-519a-5p	2.654904799	0.009893826
ExomiR-133a-5p	2.235094034	0.017677177
ExomiR-708-5p	2.983339629	0.019285256
ExomiR-6780a-5p	1.873623431	0.0251226
ExomiR-9-3p	4.012883289	0.027119896
ExomiR-137-3p	7.423805652	0.029299697
ExomiR-208b-3p	1.375012349	0.039065344
ExomiR-4732-5p	1.072666661	0.043211196
ExomiR-1304-5p	1.436695411	0.044297652
ExomiR-6875-5p	Inf	0.047985711
ExomiR-124-3p	7.164746164	0.048657967

**TABLE 4:** mTBI vs. sTBI.

Down-regulated miRs	log2FoldChange	*P*-value
ExomiR-4632-5p	−4.484323304	0.022170964
ExomiR-548al	−3.832299358	0.017681221
ExomiR-4742-5p	−2.927645265	0.027659983
ExomiR-6726-3p	−2.782014278	0.040935594
ExomiR-4479	−2.54548501	0.004788753
ExomiR-1-3p	−2.343691459	0.00368113
ExomiR-135a-5p	−2.209758632	0.029403779
ExomiR-133a-3p	−2.155987738	0.044201495
ExomiR-206	−1.772412668	0.030484354
ExomiR-3117-3p	−1.717569577	0.026369393
ExomiR-15a-3p	−1.366970802	0.020003412
ExomiR-1255b-5p	−1.331575835	0.009980007
ExomiR-6803-3p	−1.181517932	0.016763034
ExomiR-6511b-3p	−1.164545858	0.047888633
ExomiR-183-3p	−1.112352261	0.034113661
ExomiR-7975	-Inf	0.003701321
**Up-regulated miRs**	**log2FoldChange**	***P*-value**
ExomiR-382-5p	1.607886254	0.024987793
ExomiR-655-3p	1.613477731	0.027329162
ExomiR-4440	1.711868411	0.046098902
ExomiR-489-3p	1.800662954	0.046901789
ExomiR-889-3p	1.809513971	0.012725402
ExomiR-496	1.863322758	0.031121519
ExomiR-676-3p	1.867326921	0.011440932
ExomiR-136-5p	2.032218028	0.008924609
ExomiR-149-5p	2.266795239	0.023403867
ExomiR-431-5p	2.28833771	0.010259624
ExomiR-383-5p	2.319324544	0.010885954
ExomiR-1908-3p	2.428254336	0.007049684
ExomiR-154-5p	2.625506512	0.010000238
ExomiR-3622a-5p	2.762932587	0.033482363
ExomiR-549a-3p	2.8810694	0.013960583
ExomiR-4750-5p	3.924597077	0.022907773
ExomiR-211-5p	4.084582194	0.030639292
ExomiR-3934-5p	4.519222235	0.000320311
ExomiR-4714-5p	4.616080097	0.020640781
ExomiR-6789-5p	5.251332727	0.007326271
ExomiR-1262	Inf	0.000102344
ExomiR-376a-5p	Inf	0.008599005
ExomiR-6502-5p	Inf	0.015504298
ExomiR-4645-5p	Inf	0.017168725
ExomiR-4777-3p	Inf	0.020696478
ExomiR-4477b	Inf	0.041038669
ExomiR-1914-5p	Inf	0.043727216
ExomiR-6819-5p	Inf	0.048308487
ExomiR-380-5p	Inf	0.048719279

**TABLE 5:** Compared with the control group, the differential gene miR and expression shared by the mTBI and sTBI groups.

miR	Expression	miR	Expression
miR-4732-5p	Up	ExomiR-21-3p	Down
ExomiR-3154	Up	ExomiR-4433b-5p	Down
ExomiR-4745-5p	Up	ExomiR-6852-5p	Down
ExomiR-206	Up	ExomiR-199a-5p	Down
ExomiR-708-5p	Up	ExomiR-374b-3p	Down
ExomiR-137-3p	Up	ExomiR-33b-5p	Down
ExomiR-133a-3p	Up	ExomiR-301a-5p	Down
ExomiR-133a-5p	Up	ExomiR-1185-1-3p	Down
ExomiR-9-3p	Up		
ExomiR-6780a-5p	Up		
ExomiR-204-3p	Up		
ExomiR-519a-5p	Up		
ExomiR-4683	Up		
ExomiR-124-3p	Up		
ExomiR-499a-5p	Up		
ExomiR-1304-5p	Up		
ExomiR-208b-3p	Up		
ExomiR-549a-3p	Up		
ExomiR-6875-5p	Up		

**TABLE 6:** The miRs and expression shared by the control group, mTBI, and sTBI after pairwise comparison.

miR	Expression
ExomiR-206	Up
ExomiR-133a-3p	Up
ExomiR-549a-3p	Up

#### 3.3.3. GO and KEGG pathway enrichment analysis

Gene ontology and KEGG analyses of the host genes of differentially expressed miRs were conducted to predict miR functions and molecular interactions among genes.

**FIGURE 4:**
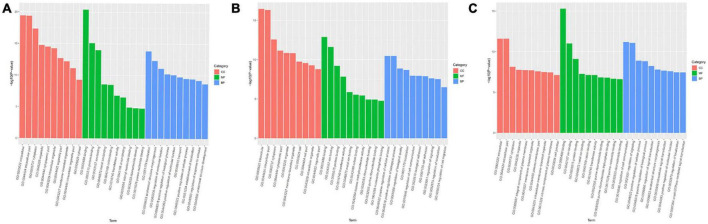
GO functional annotation results of differential miRs between mTBI, sTBI, and control. **(A)** mTBI and the control group show different gene miR GO function comment results. **(B)** sTBI and the control group’s differential gene miR-GO function annotation results. **(C)** mTBI and sTBI miR-GO function note results; CC, cellular components; MF, molecular function; BP, biological process.

Gene ontology analysis covered three domains: biological process, cellular component, and molecular function. The top 10 enriched GO terms for biological processes, cellular components, and molecular functions are shown in Figure 4. GO function annotation analyses showed that differentially expressed gene miRs are involved in the positive regulation of cellular and molecular biological processes. Compared with the control group, the mTBI group also differed in nucleotide regulation (Figure 4A). In addition, the differential gene miRs of the sTBI group are involved in the positive regulation of cellular and molecular biological processes and participate in cell adhesion and migration regulation (Figure 4B).

**FIGURE 5:**
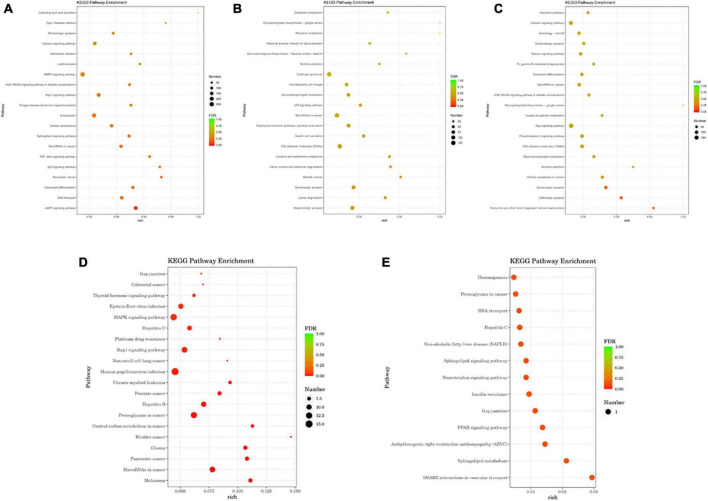
The analysis of the KEGG pathway comparing differential miRs between mTBI, sTBI, and control. **(A)** KEGG pathway analysis of the differential gene miRs between mTBI and the control group. **(B)** KEGG pathway analysis of the differential gene miRs between sTBI and the control group. **(C)** KEGG pathway analysis of the differential gene miRs between mTBI and sTBI. **(D)** Alignment of 27 miR KEGG functional annotation analyses of differential expression between control, mTBI, and sTBI. **(E)** Three miR KEGG pathway enrichment analyses showed differential expression following pairwise alignments of both controls, mTBI, and sTBI groups.

Kyoto Encyclopedia of Genes and Genomes analysis showed that differentially expressed gene miRs were related to p53 signaling pathways, calcium signaling pathways, cell adhesion molecules (CAMs), Fc gamma R-mediated phagocytosis, RNA transport, the Rap1 signaling pathway, and other pathways (Figure 5). The transforming growth factor (TGF)-β signaling pathway, cAMP signaling pathway, cellular senescence, MAPK signaling pathway, and serotonergic synapse are linked to mTBI (Figure 5A). Signaling pathways related to sTBI include *Staphylococcus aureus* infection, cholinergic synapses, and the phospholipase D signaling pathway (Figure 5B). The three miRs (ExomiR-206, ExomiR-133a-3p, and ExomiR-549a-3p) of the three groups’ KEGG pathway enrichment analysis showed that the main enrichment pathway is the soluble *N*-ethylmaleimide sensitive factor attachment protein receptor (SNARE) in vesicle transportation, gap connection, neurotrophic factor signaling pathway, RNA transportation, sphere signal pathway, peroxisome proliferator-activated receptors, and signaling channels.

#### 3.3.4. Building the PPI network of target gene

**FIGURE 6:**
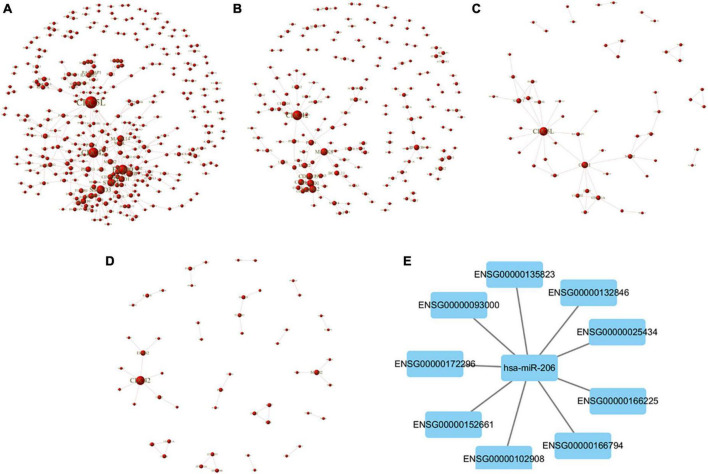
PPI network map of target genes. **(A)** Comparison of the differential expression of miRs between mTBI and control groups and a PPI network map of target genes. **(B)** Comparison of the sTBI group with the control group, screening out the differentially expressed miRs, and a PPI network map of target genes. **(C)** Comparison of the sTBI group with the mTBI group, screening out the differentially expressed miRs, and a PPI network map of target genes. **(D)** Comparison of the control, mTBI, and sTBI co-occurring 27 differentially expressed miRs to construct a PPI network map of target genes. **(E)** miR-206 and the target gene construct the PPI network diagram.

The relationship between the miR target gene was analyzed using the String database (Shrestha et al., 2019) and the PPI effect of the differential gene (Score > 0.95) was screened according to the genetic difference expression analysis results, and the PPI network was constructed. Using the Cystoscope software for visual analysis, confidence scores greater than 0.4 were obtained, and identified core modules in the network. The mTBI and control groups showed a comparative difference in expression of the miR target gene network core protein in the homology of cell division cycle 5-like (CDC5L), cell cycle divided protein 42 (CDC42), E1A binding protein (EP300), SMAD3, signal transducer and activator of transcription 3 (STAT3), and mitogen-activated protein kinase (MAPK) (Figure 6A). Compared to the control group, the sTBI group differentially expressed miRs, constructing a target gene PPI network graph core protein, mainly CDC42, MAPK (Figure 6B). In the mTBI group, the sTBI group constructing the target gene PPI network graph’s core protein was mainly CDC5L (Figure 6C).

Further alignment control screening of the 27 miR constructs for differentially expressed target genes showed CDC42 as the core protein in mTBI and sTBI (Figure 6D). PPI network plots of miR-206 and its target genes in the three miRs were differentially expressed after pairwise alignments of the control, mTBI, and sTBI groups (Figure 6E and Table 7).

**TABLE 7:** Target genes of miR206.

miR	Target	
ExomiR-206	ENSG00000025434	NR1H3
ExomiR-206	ENSG00000093000	NUP50
ExomiR-206	ENSG00000102908	NFAT5
ExomiR-206	ENSG00000132846	ZBED3
ExomiR-206	ENSG00000135823	STX6
ExomiR-206	ENSG00000152661	GJA1
ExomiR-206	ENSG00000166225	FRS2
ExomiR-206	ENSG00000166794	PPIB
ExomiR-206	ENSG00000172296	Sptlc3

## 4. Discussion

In this study, the analysis of serum exosomal miRNA expression profiles revealed eight up-regulated serum exosomal miRNAs (ExomiR-124-3p, ExomiR-137-3p, ExomiR-9-3p, ExomiR-133a-5p, ExomiR-204-3p, ExomiR-519a-5p, ExomiR-4732-5p, and ExomiR-206) and two down-regulated miRs (ExomiR-21-3p and ExomiR-199a-5) were regulated in concordance with TBI. miRs regulate gene expression and numerous other biological and pathological processes. These small RNAs have received increasing attention as potential biomarkers for detecting, identifying, classifying, and treating various diseases. Consistent with the results of our analysis, multiple studies have confirmed that multiple stable exosomal miRNAs can be detected in circulating body fluids and can change with disease progression (Martinez and Peplow, 2017; Zhou et al., 2018; Huang et al., 2020; Wu et al., 2020). O’Connell et al. determined that miR-124-3p, miR-137, and miR-9-3p had significantly higher serum levels in patients with TBI than in controls and that their sensitivity and specificity reached 90 and 100%, respectively (O’Connell et al., 2020). Using different sets of serum samples, we confirmed that the expression of serum exosomal miR-124-3p was higher in patients with TBI than in healthy controls. Vuokila et al. confirmed the chronic down-regulation of miR-124-3p after TBI, relevant to post-injury hippocampal pathologies in experimental models and humans (Vuokila et al., 2018). During the acute, subacute, and chronic phases after mTBI, microglial brain exosome miR-124-3p levels in damaged brain tissue were significantly altered, alleviating neurodegeneration and improving cognitive function (Ge et al., 2020). Additionally, one report reported that miR-9-5p may alleviate BBB damage and neuroinflammation after TBI (Wu et al., 2020). Ko et al. (2018) screened for miR expression profiles in patients with TBI by RNA sequencing. They revealed significant differences in miR-9-5a and mi-21 (*P* < 0.05) in the injured group, more accurately reflecting the heterogeneity of traumatic brain tissue injury and recovery than in the control group (Malizia and Wang, 2011; Ko et al., 2018).

They revealed significant differences in miR-9-5a and mi-21 (*P* < 0.05) in the injured group, more accurately reflecting the heterogeneity of traumatic brain tissue injury and recovery than in the control group (Malizia and Wang, 2011; Ko et al., 2018). In 2020, Ko et al. reported miR-206, which demonstrated positive predictive value for identifying injured patients vs. healthy controls. Recent studies have reported that increased miR-21-5p levels in the brain after TBI could improve neurological outcomes by alleviating BBB damage (Yin et al., 2020). Down-regulation of miR-21-3p levels in the injured brain could alleviate BBB damage by suppressing cellular apoptosis and the NF-κB-controlled inflammatory response, thereby improving neurological outcomes (Ge et al., 2019). Few studies have demonstrated that miR-133a (Dakhlallah et al., 2015) and miR-4732-5p (Sánchez-Sánchez et al., 2021) can act on ischemic cardiomyocytes to promote vasculature formation, reduce scarring, and prevent the deterioration of cardiac function. A literature search revealed that miR-199a-5p is highly expressed in myocardial tissues and is closely related to cardiac function (Tian et al., 2018; Hromadnikova et al., 2019). However, there is a lack of relevant studies demonstrating the association of miR-133a, ExomiR-519a-5p, ExomiR-4732-5p, and ExomiR-199a-5p with TBI. Consistent with these findings, the up-regulated expression of miRNAs in the TBI group was also monitored in this study, including ExomiR-124-3p, ExomiR-137-3p, ExomiR-9-3p, ExomiR-133a-5p, ExomiR-204-3p, ExomiR-519a-5p, ExomiR-4732-5p, and ExomiR-206, while ExomiR-21-3p, and ExomiR-199a-5 were down-regulated. Alternatively, we observed that miRs are potentially related to the pathophysiology of TBI and are a potential biomarker and novel target for drug therapy.

Gene ontology functional annotation analysis, and KEGG pathway analysis revealed that miRs were mainly associated with cellular aging, MAPK signaling, p53, TGF-β signaling, calcium signaling, cell adhesion molecules (CAMs), induction of neuronal injury, inflammatory mediator release, and damage to the BBB. Tao et al. (2018) showed that inhibition of MAPK pathways could mitigate microglial-mediated inflammatory responses and improve neural function. Yoong et al. (2006) demonstrated that the neurotrophic pathway can systematically and specifically regulate different miR expression levels, and the miR expression regulation involves the MAPK signaling pathway. Devoto et al. (2020) confirmed that dysregulated exosomal miRs correlate with inflammatory regulation pathways, neurological disease, and cell development. The expression levels of all the miRNAs studied may fluctuate (increase or decrease) depending on the regulation of particular biological processes involved in TBI. Among these miRNAs, hsa-miR-124-3p, ExomiR-137-3p, ExomiR-9-3p, ExomiR-133a-5p, ExomiR-204-3p, ExomiR-519a-5p, ExomiR-4732-5p, ExomiR-206, ExomiR-21-3p, and ExomiR-199a-5 are closely related to molecular function, biological processes, and cellular processes, respectively. Using bioinformatics technology analysis, it was observed that miR-199a-5, miR-133a-5p, miR-206, and ExomiR-21-3p can act on phosphoinositol metabolism, cellular senescence, and signaling pathways regulating stem cell pluripotency through SMAD3. miR-124-3p and miR-204-3p participate in the pathophysiology of patients with TBI, including activating MAPK signaling, cellular senescence, TNF signaling, and Rap1 signaling through MAPK14; miR-124-3p, miR-519a-5p, miR-9-3p, miR-204-3p, miR-4732-5p, miR-206, and ExomiR-199a-5 activate the Ras signaling pathway and Fc-mediated phagocytosis through CDC42. The miR-133a-5p and miR-519a-5p activate the cellular senescence, and TNF signaling pathways, and the VEGF signaling pathways through CDC5L.

High-throughput sequencing and bioinformatics analyses of the changes in serum exosomal miR expression profiles after brain tissue cell damage and the miR-acting target genes and signaling pathways related to TBI provide new research ideas for the study of TBI pathological mechanisms. However, this study has some limitations. First, the study is still in the initial stages of clinical research. Second, the differential expression of miRs may need to be quantitated further using PCR (Polymerase Chain Reaction). In addition, the therapeutic role of ExomiR-133a-5p a, ExomiR-519a-5p, ExomiR-4732 a-5p, and ExomiR vs. other miRNAs that are highly expressed in exosomes remains unclear. Finally, this study included a limited number of cases, which may have resulted in selection biases.

## 5. Conclusion

In this study, we extracted serum exosomal miRs from patients with TBI and identified GO functional annotation entries and KEGG pathway analysis associated with TBI. Serum exosomal miR expression profiling in patients with TBI can provide new biomarkers for the diagnosis and monitoring of TBI, which may eventually promote further research on the pathophysiological mechanism of TBI.

## Data availability statement

The datasets presented in this study can be found in online repositories. The names of the repository/repositories and accession number(s) can be found below: https://www.ncbi.nlm.nih.gov/, PRINA861423.

## Ethics statement

Ethical review and approval were not required for the study of human participants per local legislation and institutional requirements. In addition, written informed consent from the patients/participants or patients/participants’ legal guardians/next of kin was not required to participate in this study per national legislation and institutional requirements.

## Author contributions

XH and XX conceived and designed the experiments. CW, YW, YY, TY, RB, XP, FB, PL, and XX performed the experiments. XX analyzed the data. XH, XX, and XP wrote and revised the manuscript. All authors have read and approved the final manuscript.
