# Supplementary material for: Using bioinformatics technology to mine the expression of serum exosomal miRNA in patients with traumatic brain injury
Source: Front Neurosci. 2023 Apr 18;17:1145307. doi: 10.3389/fnins.2023.1145307 (PMC10151740; doi:10.3389/fnins.2023.1145307)
Supplement: Supplementary file 1 [file Table_1.DOCX]

Patient recruitment and Serum sample collection

Isolation of exosomes

miR expression in exosomes of TBI patients

identification of exosomes

TEM (Transmission Electron Microscope)

NTA (nanoparticle tracking analysis)

Western blotting

Bioinformatic analyses

Exosomal miR Library Construction and Sequencing

Filtering and miR Mapping

Differential expression analysis of Exosomal miR

Predict the target genes of miRs with significant differential expression

GO-function annotation and KEGG pathway enrichment analysis

Construct PPI network
